# From Chronic Infection to Microbial Imbalance: Rethinking Chronic Rhinosinusitis

**DOI:** 10.3390/ijms27209170

**Published:** 2026-10-16

**Authors:** Jakub Piotr Łabaz, Martyna Guth, Jakub Szymon Kaleta, Julia Kaczmarek, Malwina Kinczel, Karolina Kornak, Marta Kaczor, Anna Leszczyszyn, Karolina Dorobisz, Katarzyna Pazdro-Zastawny

**Affiliations:** 1 Faculty of Medicine, Wroclaw Medical University, Ludwika Pasteura 1, 50-367 Wrocław, Poland; martyna.guth@student.umw.edu.pl (M.G.); jakub.kaleta@student.umw.edu.pl (J.S.K.); julia.kaczmarek@student.umw.edu.pl (J.K.); malwina.kinczel@student.umw.edu.pl (M.K.); karolina.kornak@student.umw.edu.pl (K.K.); marta.kaczor@student.umw.edu.pl (M.K.); 2 Maxillofacial Surgery Department, 4th Military Clinical Hospital, Weigla 5, 50-981 Wroclaw, Poland; anna.leszczyszyn@wp.pl; 3 Department of Head and Neck Oncological Surgery, Wroclaw Medical University, Borowska 213, 50-556 Wroclaw, Poland; karolina.dorobisz@umw.edu.pl

**Keywords:** chronic rhinosinusitis, sinonasal microbiome, dysbiosis, microbial homeostasis, *Staphylococcus aureus*, *Pseudomonas aeruginosa*, biofilm, epithelial barrier, host–microbiome interactions

## Abstract

Chronic rhinosinusitis (CRS) has traditionally been viewed as a chronic inflammatory disease in which microbial factors are implicated. However, increasing evidence suggests that CRS may be better understood as a disruption of sinonasal microbial homeostasis rather than as a chronic infection caused by a single pathogen. This review summarizes current knowledge on healthy sinonasal microbiome composition and function as well as microbial alterations in CRS. Particular attention is given to dysbiosis involving potentially pathogenic microorganisms, including *Staphylococcus aureus* and *Pseudomonas aeruginosa,* as well as biofilm formation and its interactions with the epithelial barrier and host immune response. We also discuss host-related, therapeutic, hormonal, and environmental factors that may contribute to microbial imbalance. Current evidence does not clearly establish whether dysbiosis is a cause or consequence of chronic inflammation. Based on the evidence synthesized here, we propose that CRS may be understood as a dynamic, bidirectional interaction between microbial imbalance and chronic inflammation, whereby microbial alterations may contribute to epithelial and immune dysregulation, while changes in the host environment influence the sinonasal ecosystem. By integrating these microbiological and host-related perspectives, this review provides a boarder framework for understanding CRS pathophysiology and guiding future research into individual patterns of sinonasal microbial homeostasis.

## 1. Introduction

### 1.1. Definition and Classification of Chronic Rhinosinusitis

Chronic rhinosinusitis (CRS) is a heterogeneous disease characterized by inflammation of the nasal and paranasal sinus mucosa persisting for at least 12 weeks. CRS significantly impairs patients’ quality of life through a range of local and secondary symptoms while also generating direct and indirect healthcare costs [1,2].

Since 1980, an increase in the incidence of CRS has been observed. A systematic review and meta-analysis of observational studies conducted between 1980 and 2021 included 28 studies with a combined total of over 237 million participants, yielding 11,342,923 cases of CRS [3]. The meta-analysis determined the global pooled prevalence of CRS to be 8.71% (95% CI: 6.69–11.33; 20 studies), while the prevalence of CRSwNP was 0.65% (95% CI: 0.56–0.75; 4 studies) [3]. The prevalence of these conditions is higher in adults than in children and increases in the presence of predisposing factors such as smoking, obesity, asthma, eczema, diabetes, and a deviated nasal septum [3].

CRS can be divided into two phenotypes: CRS with nasal polyps (CRSwNP) and CRS without nasal polyps (CRSsNP). Differences between the phenotypes also extend to clinical aspects. The disease course is significantly more severe in CRSwNP, as this phenotype is associated with a greater need for surgical interventions and pharmacological treatments—primarily involving antibiotics or glucocorticosteroids. Additionally, CRSwNP is characterized by a higher prevalence of comorbid asthma and a higher rate of recurrence compared to CRSsNP [4,5]. Nevertheless, both phenotypes are characterized by structural remodeling of the nasal and paranasal sinus mucosa [4].

The pathophysiology of CRS encompasses three main patterns of inflammatory response, designated as types 1, 2, and 3. However, this division represents a simplified model of the dominant immunological mechanisms and, consequently, should not be viewed as a classification of mutually exclusive endotypes. Depending on the patient, inflammatory pathways may coexist and overlap, leading to mixed endotype profiles such as types 1/2, 1/3, 2/3, and—less frequently—1/2/3. Assigning CRS to a specific endotype indicates the dominant type of immune response rather than the exclusive presence of a single inflammatory mechanism [6]. The aspect of overlapping inflammatory pathways should also be considered regarding the relationship between the microbiota and the immune response in CRS. Dysbiosis and changes in microbiota composition do not necessarily correspond to a single endotype; they may be associated with the activation of co-existing inflammatory pathways [7]. Type 1 is significantly more common in women and is associated with Th1 cells, which act via cytokines such as IL-12, IL-2, TNF-β, and IFN-γ. Its function involves defense against intracellular pathogens and the induction of cytotoxic and local inflammation [8,9,10]. Type 2—associated with Th2 cells and cytokines IL-2, IL-4, IL-5, and IL-13—is the best-characterized inflammatory pattern in CRS and predominates particularly in patients with CRSwNP in Western populations. It is linked to eosinophilic infiltration and elevated IgE levels. Characteristic features include the presence of polyps, co-existing asthma, allergic mucin, and loss of smell. Type 3 inflammation operates via Th17 cells, which secrete IL-17, IL-22, and IL-23. These cytokines lead to the recruitment of neutrophils and macrophages, resulting in the presence of pus in the sinuses [8,9]. It has been observed that the disease is more resistant to treatment in patients with Type 1 and Type 3 inflammation [11].

The global prevalence of inflammatory endotypes varies by region. In Western Europe and the Americas, CRSwNP characterized by a type 2 immune response predominates, whereas in the East (East Asia and Japan), a Th1/3 profile with neutrophilic infiltration is more common. However, recent years have seen an increase in Th2-driven CRSwNP in Asian countries [4,12]. This carries specific clinical implications that must be considered during diagnosis and the selection of appropriate treatment.

It is worth noting that phenotypes are not specific to endotypes, and distinct pathogenic mechanisms are frequently observed within a single phenotype. Nevertheless, because endotyping provides information on biochemical mechanisms, it is often more significant than phenotyping [13]. Consequently, the development of improved endotyping methods can help advance and expand the use of biologics for the treatment of CRS associated with type 2 inflammation—and potentially type 1 or 3 inflammation in the future [14]. The relationship between biologic therapy and the paranasal sinus microbiome remains poorly understood—in contrast to methods directly targeting microbiome modulation, which are increasingly being applied in clinical trials and are discussed in greater detail in Section 5. Data regarding the impact of biologics on the composition and function of the sinus microbiota are very limited—at both preclinical and clinical levels—despite the growing importance of these drugs in the treatment of CRS. Furthermore, it remains unclear whether the baseline microbiota profile or the degree of dysbiosis influences the response to biologic therapy. Defining the relationship between biologic therapy and the microbiome therefore represents a significant research gap that warrants further study [15]. A summary of the current knowledge on inflammation endotypes is provided in Table 1.

### 1.2. Etiopathogenesis

In recent years, the understanding and concepts regarding the pathophysiology of CRS have evolved. Earlier approaches focused on impaired sinus ventilation and patency, as well as on mechanisms involving bacterial infections and toxins. Following the identification of CRS endotypes in 2016, a mucosal concept was proposed, focusing on functional and structural abnormalities in the sinus mucosal epithelial cells [16]. Physiologically, the nasal cavity and sinuses are protected by an epithelial layer. This layer acts as a physical and immunological barrier and facilitates mucociliary clearance—an innate respiratory defense mechanism that maintains mucosal homeostasis, removes bacteria and toxins, and preserves water–electrolyte balance [4,17]. Disruption of the epithelial barrier in the sinuses and nasal cavity can increase exposure to antigens, airborne pathogens, and particulate matter. In the context of a dysregulated immune response, this elevates the risk of chronic inflammation [18]. Furthermore, compromised epithelial barrier integrity affects the efficacy of CRS treatments, including glucocorticosteroids and biologics [16,17,19,20]. In patients with CRS, the baseline ciliary beat frequency shows reduced responsiveness to environmental stimuli. Due to impaired clearance, bacteria may begin to colonize the epithelium and form a biofilm [20]. Impairments in mucociliary clearance and epithelial barrier integrity affect the local microbial environment of the sinus mucosa. Alterations in the microbiota can influence epithelial barrier function and the host immune response, thereby perpetuating chronic inflammation. Additionally impaired clearance promotes mucus stasis, biofilm formation, and microbial persistence, contributing to the development of dysbiosis. The interrelationships between epithelial dysfunction, the microbiota, and inflammation can reinforce one another; however, the directionality and relative importance of these individual processes remain not fully elucidated [21].

The epithelium of the nasal cavity and sinuses is heterogeneous in terms of cell composition. It contains basal cells (BCs), goblet cells, submucosal cells, ciliated cells, glandular cells, and secretory cells. Ionocytes and myoepithelial cells have also been identified. Under physiological conditions, BCs are capable of self-renewal, as well as differentiation into other epithelial cell types. In the course of CRS, a significantly increased number of undifferentiated BCs is observed in the epithelium. Their proliferation is driven by Th2 response mediators—specifically, IL-13 and enterotoxin B (secreted, for instance, by *S. aureus*) [16,17].

Epithelial–mesenchymal transition (EMT) is a significant process occurring in the respiratory epithelium during chronic inflammation associated with CRS. EMT enables the transformation of a polarized epithelial cell into a mesenchymal cell, a process accompanied by the loss of intercellular junctions (TJs); for instance, in CRSwNP, reduced levels of proteins such as occludin, claudin-1, DSG1, DSG2, and ZO-1 are observed [22]. While EMT is physiologically essential for epithelial repair, CRS is characterized by enhanced EMT associated with a continuous cycle of epithelial cell injury and repair. EMT in CRS is induced by various factors, including allergens, hypoxia, pathogens, tobacco smoke, HIF-1α, and type 2 cytokines. Consequently, EMT contributes to tissue fibrosis and the persistence of the disease, creating a vicious cycle mechanism [16,22].

In CRSwNP, the levels of epithelial antimicrobial proteins are significantly lower than in healthy individuals. These proteins are responsible for eliminating pathogens and protecting the normal microbiome. They include lysozyme; beta-defensins; palate-, lung-, and nasal-epithelium clone proteins; S100A7 (psoriasin); and S100A8/9 (calprotectin) [14]. In CRSwNP, conversely, elevated levels of proteins contributing specifically to polyp formation are observed—namely pendrin, an ion transporter that increases mucus production, and Muc5AC, a type of mucin [14,18].

CRS is also influenced by immune system dysregulation. CRS endotypes are described in detail above, in Section 1.1. CRSwNP is characterized predominantly by type 2 inflammation with tissue eosinophilia. Levels of eosinophil cationic protein, eosinophil chemotactic proteins, IL-5, IL-13, IL-36, thymic stromal lymphopoietin (TSLP), basophils, and mast cells are elevated. Exposure to environmental stimuli prompts epithelial cells to secrete various inflammatory cytokines and chemokines, such as IL-25, IL-33, IL-1β, TNF-α, and CXCL8. In turn, TSLP—by stimulating the release of IL-4, IL-5, and IL-13—activates dendritic cells, thereby promoting Th2 cell differentiation [18,23]. Enhanced interaction between epithelial cells and immune cells establishes a positive feedback loop that amplifies eosinophilic and neutrophilic inflammation [23]. It is worth noting that epithelial cells possess an “inflammatory memory” function, which predisposes CRS to recurrence following surgical intervention. The primary cytokines responsible for this memory are IL-4 and IL-13—components of type 2 inflammation—which explains the higher rate of disease recurrence observed in CRSwNP [18,23].

With recent developments in medical knowledge, the microbiome is being potentially recognized as an important part of CRS pathophysiology, with persistence of inflammatory processes linked to particular bacterial colonization patterns [7]. Under physiological conditions, various bacterial species inhabit the respiratory mucosa, and recent evidence also indicates the presence of viruses and fungi [7,24,25]. Commensal organisms play a role in eliminating pathogens and regulating a proper host immune response. While potentially pathogenic organisms may be present in low numbers in a healthy sinus, shifts in the general composition of the microbial community may trigger their excessive proliferation, which, in turn, can fuel inflammatory processes characteristic of CRS [7]. Dysbiosis can also create conditions under which otherwise benign microorganisms become invasive. Furthermore, anaerobic conditions within the sinus cavities favor the growth of anaerobic bacteria in patients with CRS [24,25]. Changes in the composition of the sinus microbiome associated with CRS, as well as pathogen virulence mechanisms, are discussed in detail in Section 3.

It is worth noting that the distinct nasal and sinus microbiome profiles observed in patients with CRS may reflect not only the disease state itself but also the effects of medications commonly used in its management, particularly antibiotics and corticosteroids [26,27,28]. Therefore, when interpreting microbiome-related associations, treatment exposure should be considered a potential confounding factor (more on this in Section 5.1).

A further significant factor influencing the occurrence and course of CRS is genetic predisposition. Due to the multifactorial etiology of the disease, it is difficult to pinpoint specific genes responsible for its development; however, certain genetic abnormalities can increase susceptibility to CRS or lead to its secondary development [29,30,31,32]. An example is a mutation in the CFTR gene, which is responsible for cystic fibrosis. An abnormal chloride channel structure—or its complete absence—can contribute to impaired mucociliary clearance; polyp formation; and the production of thick, viscous mucus that creates an ideal environment for pathogen growth. Thus, CRS may occur as a condition secondary to cystic fibrosis, although this complication arises in only a small percentage of cases [29].

Another area of genetic research in CRS focuses on type 2 taste receptors (T2Rs), which have been identified not only on the tongue but also in the sinus epithelium. T2R activation increases intracellular calcium levels and enhances the local production of nitric oxide (NO), a molecule involved in airway defense mechanisms and the regulation of inflammatory responses [30]. Prospective clinical trials have shown that reduced NO levels can promote inflammation, whereas individuals with the PAV/PAV genotype (referred to as “supertasters”) exhibit higher NO concentrations, a factor associated with a lower incidence of sinusitis [29,30].

CRS can also occur secondary to genetically determined ciliary dysfunction, such as primary ciliary dyskinesia (PCD), which is associated with mutations in as many as 40 genes—most commonly, DNAH5 and DNAI1. Abnormal ciliary structure and function impair mucociliary clearance, thereby promoting the development of CRS, although no definitive link has been established between the severity of CRS and a specific type of ciliary defect [29,31].

Apart from ciliary dysfunction, genetic studies also point to numerous other genes potentially associated with CRS, including those involved in immune responses, tissue remodeling, and the regulation of inflammation—such as AOAH, TLR2, genes encoding lactoferrin, KCNQ5, KCNMA1, LAMA2, and LAMB1 [29,32]. Associations have also been demonstrated between the occurrence of CRS and polymorphisms in genes related to type 2 responses, TGF-β, MMPs, PAI-1, arachidonic acid metabolism, and cytokines [29,32].

Environmental factors play a significant role in the etiopathogenesis of CRS. These include, among others, tobacco smoke, air pollution, allergens, toxins, microbiological agents, and occupational exposures [33]. Among these, the impact of tobacco smoke is the best understood; both active smoking and passive exposure to tobacco smoke are associated with an increased risk of CRS in adults and children, respectively [33]. Detailed information regarding the impact of air pollution and tobacco smoke on the development of CRS is provided in Section 4.3 of this review.

Harmful agents associated with workplace exposure include animal dander, fish, crustaceans, latex, pharmaceutical products, reactive disinfectants, and metal fumes [33]. Data regarding occupational exposure are more limited, although available studies indicate an increased risk of CRS among individuals exposed to gases, dusts, chemical fumes, and other airborne contaminants in the workplace [34,35]. Chronic exposure to these factors can promote irritation and inflammation of the mucous membrane [35].

Taken together, these findings highlight the multifactorial nature of CRS and the complex interplay between microbial, epithelial, immune, genetic, hormonal, and environmental factors. Previous studies and reviews included in the present review have provided important insights into individual aspects of the sinonasal microbiome, including microbial community structure and diversity, dysbiosis, microbial interactions, pathogen-associated mechanisms, therapeutic perspectives, and methodological aspects of microbiome research [25,36,37,38,39,40,41,42]. However, these microbiological dimensions have generally been addressed separately rather than integrated into a single comprehensive framework. The present review builds on this body of evidence by integrating these previously addressed microbiological perspectives and examining them alongside broader factors that may shape the sinonasal ecosystem, including epithelial barrier and mucociliary dysfunction [4,17,20], inflammatory endotypes [4,8,9], genetic susceptibility [29,31,32], hormonal signaling [43,44,45,46], and environmental exposures [33,35,47]. The novelty of the present review therefore lies not in introducing an entirely new individual mechanism but in integrating these previously dispersed areas of evidence into a single host–microbe–environment framework for CRS. In this framework, microbial imbalance and host-related processes are considered as potentially bidirectional and mutually influencing while recognizing that the available evidence does not establish a universal causal sequence [21]. The present review therefore considers the sinonasal microbiome not as an isolated compartment but as an ecosystem continuously influenced by local host factors as well as systemic, hormonal, genetic, and environmental conditions.

### 1.3. Materials and Methods

A comprehensive narrative review of the current evidence concerning the role of the sinonasal microbiome and microbial dysbiosis in chronic rhinosinusitis (CRS) was conducted using the Scopus, PubMed, and Web of Science databases. The literature search and selection process was conducted from November 2025 to August 2026, with the final literature screening and manuscript revision completed at the turn of August and September 2026. The search strategy combined terms related to chronic rhinosinusitis and its phenotypes, including chronic rhinosinusitis with nasal polyps (CRSwNP) and without nasal polyps (CRSsNP), with terms describing the sinonasal microbiome and microbiota, microbial dysbiosis and homeostasis, bacterial pathogens and biofilms, epithelial barrier function, host-microbiome interactions, inflammatory and immunological mechanisms, environmental and hormonal factors as well as the gut-airway and nose-brain axes. The following terms were used in the title and abstract searches: “chronic rhinosinusitis”, “CRS”, “chronic rhinosinusitis with nasal polyps”, “CRSwNP”, “chronic rhinosinusitis without nasal polyps”, “CRSsNP”, “sinonasal microbiome”, “nasal microbiota”, “dysbiosis”, “microbial homeostasis”, “*Staphylococcus aureus*”, “*Pseudomonas aeruginosa*”, “biofilm”, “epithelial barrier”, “host-microbiome interaction”, “air pollution”, “sex hormones”, “gut-airway axis”, and “nose-brain axis”.

The review primarily considered original research articles and relevant clinical or consensus documents, while systematic reviews and narrative reviews were also included when relevant to the scope of the present review. Publications were selected according to their relevance to at least one of the 6 major thematic domains (biological, microbiological, immunological, environmental, hormonal or clinical) addressed in the review. Publications that did not directly contribute to the scope of the review, including those without significant evidence concerning the sinonasal microbiome, microbial dysbiosis or homeostasis, microbial virulence and biofilms, epithelial and mucociliary function, host immune responses and inflammatory endotypes, environmental or hormonal modulation, or gut–airway interactions, were not considered for inclusion. Conference abstracts, conference proceedings, letters to the editor, commentaries, and other publication types that did not provide sufficiently detailed primary or synthesized evidence relevant to the scope of the review were also excluded. Only articles written in English were included.

No strict publication-date restriction was applied, as selected older studies were retained when they provided foundational evidence or remained directly significant to the mechanisms discussed in the present review. In addition to database searches, relevant publications were identified through examination of the reference lists of key articles and reviews, allowing identification of primary studies and other pertinent literature not necessarily retrieved through the initial keyword searches.

Following the literature search and selection process described above, a total of 233 publications were cited in the final review.

## 2. Healthy Sinonasal Microbiome

With recent developments in scientific methods and increased understanding of the pathophysiology of diseases, it has become increasingly evident that the microbiome—defined as the entirety of microorganisms present within a specified habitat, including their genome and metabolytes—plays a critical role in balancing between the thin veil of disease and health in the human body. The relationship serves both involved parties—the microbiome is allowed a hospitable environment, whereas the human innate immune system is stimulated in preparation for encounters with possibly harmful exogenous factors. Over the years, multiple studies have shown what the physiological sinonasal microbiota mainly consists of. The identification was possible by using 16S rRNA sequencing, shotgun metagenomics or whole-genome deep sequencing. It has been revealed that *Firmicutes*, *Actinobacteria* and *Proteobacteria* phyla, as well as *Fusobacteria* and *Bacteroidetes* occurred in comparatively smaller abundance. On the genus level, *Corynebacterium*, *Staphylococcus*, *Propionibacterium*, *Cutibacterium*, *Dolosigranulum*, *Moraxella*, *Lactobacillus*, *Streptococcus* and *Acinetobacter* are the most prevalent [36,37,48,49,50,51,52,53,54,55,56,57,58].

For years, bacterial identification was widely performed using classical culture methods [59]. However, laboratory analysis of bacterial composition often requires specific growth media, and cultivating certain bacteria is not always possible [60,61]. Therefore, gene sequencing methods, which are not culture-based, are also considered for such analyses. For instance, amplicon sequencing allows for the identification of bacterial genera (and sometimes species), while shotgun metagenomics enables analysis at the species and strains levels, as well as the detection of fungi and viruses. However, there is a risk of sample contamination while choosing sequencing methods, which can lead to false results [62]. Furthermore, errors and gaps in databases can result in the misclassification of bacteria, so further standardization of these methods is required [63].

Despite the fact that most studies focus solely on bacteria in regard to the human microbiome, there are also other inhabitants of the sinonasal mucosa, such as fungi and viruses. Although a previous analysis failed to detect any fungi genetic material in the swab samples of middle meatus mucosa collected from the healthy control group [64], a number of other works have positively identified the presence of fungi in the human nasal mucosa using shotgun metagenomics and internal transcribed spacer (ITS) sequencing. This suggests that they play a significant role in stabilizing this environment [49,65]. Within the sinus mycobiome, several species of the genus *Malassezia* (such as *M. restricta*) stand out, playing a commensal role in the sinuses [36,66]. On the other hand, some studies have shown detectable viral traces in the human nose, which was possible through the use of Polymerase Chain Reaction (PCR), Real-Time Polymerase Chain Reaction (RT-PCR), and Enzyme-Linked Immuno Sorbent Assay (ELISA). Importantly, the two most commonly isolated species—Epstein–Barr virus (EBV) and human herpesvirus 6 (HHV-6)—were prevalent in both healthy and CRS patients, which implicates a more complex role of viruses in chronic sinonasal inflammation [67,68]. The full scope of interactions between the bacterial, fungal and viral organisms remains largely unknown, and their role in maintaining the physiological sinonasal microbiome should be further examined.

As we get a better understanding of the human nasal microbiome, some of its specific traits are becoming increasingly visible. Numerous studies using 16S rRNA sequencing and qPCR for analysis have concluded that there is significant variability between people in terms of the composition of the sinonasal microbiome [36,50,51,54,55,58,69,70]. Furthermore, longitudinal studies carried out in recent years suggest that the intra-person nasal microbiome remains relatively stable over time periods [36,58,69]. These results contribute to an inference: a characteristic feature of the paranasal sinus ecosystem is the striking dichotomy between its high inter-individual variability and remarkable intra-individual stability, which gives this ecosystem a ‘bacterial fingerprint’ unique to each person. This suggests that ‘health’ is not a single, universal microbial profile but a personalized homeostatic balance.

Microbiome studies have allowed us to observe that sinonasal microorganisms are not bystanders. Instead, they engage in a sophisticated network of multilateral interactions—not just among themselves but also with the mucosa. Commensal colonizers have exhibited potential to act against possible pathogens in both active and passive manners. Passively, they compete to establish themselves in a niche, which includes appropriate space, access to nutrients and surface receptors, as well as beneficial interactions between other inhabitants. On the other hand, active rivalry is possible due to a number of secreted antimicrobial substances.

This interplay can be demonstrated in several examples. *Dolosigranulum pigrum*, a common nasal commensal, has the potential to inhibit the growth of *Staphylococcus aureus* [71], not only a frequent opportunistic pathogen, but also a species often associated with changes in the microbiota in CRS [52]. This negative correlation is enhanced when *D. pigrum* coexists with *Corynebacterium pseudodiphtheriticum/propinquum*, with which *D. pigrum* also correlates positively [58,72]. Additionally, coculture of the two aforementioned species had a negative effect on the growth of *Streptococcus pneumoniae*, yet another pathogen common in CRS [72]. Moreover, Man et al., based on the analysis of nasopharyngeal swabs obtained from participants, observed that the presence of *C. pseudodiphtheriticum/propinquum* and *D. pigrum* in the nasopharynx correlates negatively with lower respiratory tract infection in young children [73]. Again, these two species are linked to better recovery after sinus surgery in CRS, as well as less exacerbated clinical course of CRSwNP [52]. Kanmani et al. noticed that infant mice, upon nasal administration of *C. pseudodiphtheriticum/propinquum*, displayed increased resistance against primary infection with respiratory syncytial virus (RSV), followed up by a secondary infection of S. pneumoniae [74]. Another typical sinonasal colonizer, *Corynebacterium accolens*, has exhibited antimicrobial properties in in vitro studies against both *S. aureus* [75,76] and *S. pneumoniae* [77]. Menberu et al. observed that strains of *C. accolens* cultured from non-CRS patients inhibit the growth of both MSSA and MRSA strains isolated from the sinonasal mucosa of CRS patients, which is most likely caused by an uncharacterized protein produced by the commensal microorganism [75]. Bomar et al. concluded that an enzyme produced by *C. accolens* releases free fatty acids from a sample host surface in vitro, which, in turn, have antipneumococcal activity [77]. Furthermore, in vivo and in vitro studies by Ramsey et al., as well as in vitro studies by Chalermwatanachai et al., have pointed at an interesting trait possessed by a number of commensal *Corynebacterium* species—their large abundance in an environment pushes *S. aureus* towards a commensal lifestyle instead of a pathogenic one, via modified expression of the *S. aureus* quorum sensing-controlled accessory gene regulator (agr) genetic locus, which has a role in both colonization and virulence [78,79]. *Cutibacterium acnes* (formerly *Propionibacterium acnes*), widely associated with acne, also seems to have a role in the complex web of dependencies in the pathogenesis of CRS. Li et al. observed, in a murine model, that propionic acid produced by *Parasutterella* genus drives down inflammation via an indirect inhibitory effect on the survival and proinflammatory functions of M1-like macrophages [80]. Propionic acid is a key metabolite of *C. acnes* [81]; thus, it may exhibit similar properties. A bacteriocin produced by *C. acnes*, cutimycin, has also been under the scientific spotlight lately, with reported antifungal and antibacterial properties, as well as a possible modulating effect on immune reactions to pathogenic microorganisms [38]. In an in vivo study, Claesen et al. demonstrated that cutimycin is produced in hair follicles, while an in vitro study revealed that it has antibacterial potential in regard to MRSA [82]. Conversely to this evidence, Hardy and Merrell found that *C. acnes* engages in a non-antagonistic relation with *S. aureus* via the Christie–Atkins–Munch–Petersen (CAMP) factor, released by the former, which increases the activity of the *S. aureus* beta-hemolysin toxin. This, in turn, has been linked to higher chances of *S. aureus* colonization and promotion of invasive disease [83]. Moreover, *C. acnes* benefits from the coexistence of *S. aureus* thanks to the synthesis of staphyloferrins, which can become xenosiderophores for a number of sinonasal commensals and stimulate their growth as an accessible source of iron [84]. In summary, scientific evidence shows that the relationship between *C. acnes* and *S. aureus* is sophisticated and remains to be further studied.

*S. aureus*, despite its pathogenic potential, is frequently isolated from nasal mucosa samples of healthy people by culturing, shotgun metagenomics and 16S rRNA sequencing [50,85]. In an in vitro study, Zhao et al. found that *C. pseudodiphtheriticum/propinquum* profits from staphyloferrin secretion either via a ‘shared labor’ relation or through one-sided ‘siderophore cheating’ [84]. Additionally, Schwartz et al. linked the lower bacterial load of *S. aureus* to increased anti-inflammatory reaction mediated by IL-10, combined with a decrease in TNF—often associated with pro-inflammatory response [86]. In an ex vivo study using nasal tissue, Altunbulakli et al. observed that *S. aureus* colonization in healthy subjects was positively associated with the expression of intercellular adhesion factors and overall improved epithelial barrier function [87].

It is important to mention that *Lactobacillus* species also influence the maintenance of balance in the sinus environment, and these homeostatic properties are currently used therapeutically in the treatment of CRS. The *Lactobacillus* genus, although generally not found in great abundance, has been previously described as a commensal of the upper respiratory tract [88]. When analyzing samples from healthy and CRS patients, using 16S rRNA sequencing, Boeck et al. found that the most prevalent species are *L. rhamnosus*, *L. casei*, *L. sakei* and *L. plantarum* and that their number was lower in CRS samples [89]. Interestingly, the relative abundance of lactobacilli seems to correlate positively with mucosal integrity in CRSwNP patients via modulating expression of intercellular junction proteins [90]. One *L. casei* strain was studied for its traits that allow it to inhabit the specific environment of the human nose. The strain exhibited increased oxidative-stress tolerance, which contributed to the production of a catalase, as well as enhanced adherent properties thanks to adhesin proteins and fimbriae-like structures [87]. Tight adherence counteracts nasal clearance, which is particularly advantageous for nasal applications. Having that in mind, the same *L. casei* strain was investigated as a possible nasal probiotic. In an in vitro study, Martens et al. [89] found that *L. casei* acts on Toll-Like Receptor 2 (TLR2) to stimulate a correct immune response and reorganize intercellular tight junctions to increase epithelial integrity in air–liquid interphase cellular cultures based on CRSwNP samples. Additionally, the strain can restore IL-4-mediated epithelial dysfunction via an indirect effect on the NF-κB signaling pathway, which is critical in modulating the actin cytoskeleton. This beneficial effect has been demonstrated both in vitro and in vivo. On the other hand, an in vitro study analyzing the effects of an *L. sakei* strain found no protective effect on the sinonasal epithelium [89]. Despite these results, *L. sakei* has been previously attributed probiotic functions such as mitigating inflammatory reactions in colitis mice [91] and improving the clinical picture of obesity based on a murine model [92] and has exhibited anti-biofilm and antioxidative properties [93]. This contrast signifies the need for further research on the various effects of the *Lactobacillus* genus on the nasal epithelium.

In addition to the complex interaction of sinus bacteria, as described above, they also take part in shaping both metabolic and structural aspects of the nose and sinuses. These functions manifest in various ways, from regulating the integrity of epithelial barrier through metabolizing leftover polysaccharides and mucosal ingredients [38,94].

## 3. Alterations to the Microbiome in Chronic Rhinosinusitis

As outlined in EPOS 2020, CRS is a diverse medical condition that encompasses a number of endotypes and phenotypes, each with their distinctive characteristics, different underlying pathologic processes and varying therapeutic prognoses (more on this in Section 1.1) [95]. Changes in the sinonasal microbiome have been suggested to play a critical role in CRS pathogenesis. Available scientific evidence points at two possible concurrent pathways in which the microbiome may contribute to the clinical manifestations of CRS: alterations to the diversity and stability of the sinus microbiota, as well as localized domination of pathobionts—mainly, *Pseudomonas aeruginosa* and *Staphylococcus aureus*. These two species, among others, may be behind various unwanted processes, for example, pathologic epithelial remodeling, defective mucociliary clearance and dysfunction of the mucosal barrier (more on this later in the article) [96]. As the role of fungi and viruses in the pathogenesis of CRS is largely unexplored and requires further research, further discussion focuses solely on bacteria.

### 3.1. Chronic Rhinosinusitis as a Result of Reduced Bacterial Diversity and Stability

The dysbiosis hypothesis implicates that changes to the microbiome entailing both increasing abundance of opportunistic pathogens and the depletion of pivotal commensal species may contribute to numerous chronic diseases, including CRS [97]. In regard to CRS, bacterial dysbiosis is usually described in comparison to a healthy cohort and is often distinguished by lower diversity and stability, increased overall bacterial abundance, and the two traits mentioned above at the same time. Nevertheless, standardized criteria for this imbalance have not been defined so far [37,39].

Numerous works have emphasized the loss of abundance in *Corynebacterium* and *Dolosigranulum* taxa—most notably, in CRSwNP. As previously mentioned, these bacteria might have protective effects on the nasal epithelium and exhibit anti-inflammatory properties [37,50,98,99]. Additionally, samples from CRSwNP patients had greater abundance of *Staphylococcus* taxa, including *S. aureus* [50,52,54,78]. It is noteworthy that Chalermwatanachai et al., using 16S rRNA sequencing for microbiota analysis, reported a greater relative abundance of *Haemophilus influenzae* in patients with CRSwNP compared to the control group and suggested a role for this species in promoting pro-inflammatory reactions that may be involved in the development of nasal polyps [78]. In comparison to CRSwNP, a different set of commonly promoted bacterial taxa was observed in CRSsNP. Lal et al., also using 16S rRNA sequencing, noticed that mucosal samples from CRSsNP patients were enriched in *Streptococcus*, *Haemophilus*, and *Fusobacterium* taxa, among other facultative and obligatory anaerobes, when paralleled with the healthy cohort [54]. These microorganisms may locally promote pathologic processes. For example, *Fusobacterium* spp. are commonly associated with purulence in CRSsNP [100]. Overall, Liu et al. pointed out that even among healthy cohorts, nasal microbiome profiles with greater abundance of potentially pathogenic species have lower nasal colonization stability [58].

### 3.2. The Role of Pseudomonas aeruginosa

*P. aeruginosa* is commonly mentioned as a bacterium capable of secreting a wide range of toxic substances exhibiting effects that might contribute to pathologic processes associated with CRS [40,101]. It has been isolated more often in patients with CRS compared to healthy subjects and is generally linked with treatment-resistant CRS [102,103,104], reflecting the contribution of numerous virulence factors, as well as biofilm formation, a trait repeatedly connected with high antibiotic resistance [101,104]. When investigating the effect of *P. aeruginosa* infection on airway organoid models, Boeck et al. found that the infected organoids had greatly downregulated gene sets associated with overall cilia function and structure, including their movement, assembly and organization, which suggests *P. aeruginosa* as a pathogen responsible for ciliary impairment [105]. Pyocyanin, a pigment with oxidizing capabilities and a toxic secondary metabolite of *P. aeruginosa*, has been thoroughly examined over the years. It is now known that pyocyanin acts destructively and as a pro-inflammatory on human the epithelium in multiple ways. As an oxidizing agent, it can induce overproduction of reactive oxygen species (ROS) [106], which have been found to disrupt ciliary function [107]. In detail, pyocyanin-induced ROS damage mitochondria, which, in turn, leads to ATP deficit [85], a substrate crucial to ciliary movement via dyneins, motor proteins responsible for ciliary beating [108]. A specific ROS, hydrogen peroxide, is also responsible for activating protein kinase C (PKC), which can indirectly reduce ciliary beat frequency [109]. Furthermore, ROS can disrupt the epithelial barrier acting on intracellular actin filaments [110], as well as intercellular tight junctions and adherens junctions, which leads to deficient mucosal immunity [111]. ROS overabundance also contributes to the production of IL-8, a strong neutrophil attractor. It has been suggested that pyocyanin can influence excessive neutrophil extracellular trap (NET) formation. Although NETs are important immunologic mechanisms, their prolonged presence may cause persistent inflammation, which, in turn, contributed to the release of other pro-inflammatory compounds, as well as ROS, inevitably setting the ground for a feedback loop of chronic inflammation. Prolonged NETosis can also disrupt ciliary function and thicken mucus [112,113]. Moreover, Zhu et al. reported that pyocyanin can selectively reduce phagocytic capabilities of macrophages in vitro [114]. Additionally, the pigment regulates cytokine release, making it prolonged and of greater magnitude, which pushes a healing-associated cytokine profile, often associated with acute inflammation, into a pro-inflammatory one, associated with chronic processes [115,116]. A number of studies have shown that long-term pyocyanin exposure is correlated with goblet cell metaplasia and hyperplasia, along with mucus hypersecretion [117,118,119]. Moving away from pyocyanin, Kao et al., based on a study using nasal epithelial cells obtained from participants, observed the detrimental effect of serine proteases released by both *P. aeruginosa* and neutrophils on the coherence and permeability of the epithelial barrier [120]. Another study, conducted in vitro, reported adverse effects of *P. aeruginosa*-associated elastase activity on the proteins involved in intercellular epithelial junctions. The barrier degradation was dose-dependent, and elastase activity visibly correlated with CRS disease severity (based on CT-scan scores) [121]. Moreover, these enzymes are linked to delayed epithelial repair [122,123].

It is noteworthy that scientific evidence points out that the species can change its form between planktonic and biofilm and, when doing so, change its phenotype, including the modulation of virulence and persistence factors [103,124]. Jurado-Martín et al. concluded that strains associated with chronic infections can upregulate persistence factors, for example, via a reduced growth rate, lipopolysaccharide (LPS) modification and production of alginate-associated biofilms in exchange for the giving up of virulence potential [124]. Previous works indicate that alginate has the ability to disrupt immune signaling via calcium chelation [125], which may reveal itself in the form of excess TNF-α production paired with downregulated IL-6 release [126]. In accordance with these findings, Tuli et al., based on an in vitro study, observed that in comparison with a negative control, biofilm-secreted exoproteins had little effect on IL-6 production, whereas planktonic-secreted exoproteins in the same concentration greatly elevated IL-6 production, hinting at its pro-inflammatory potential [103]. Furthermore, Biswas et al. analyzed mucus samples from the sino-nasal cavities of participants and observed a strong positive correlation between *P. aeruginosa* and the activity of Toll-Like Receptors (TLRs) 2/1 and 4 [127], which are known to stimulate the release of pro-inflammatory cytokines such as IL-6 and IL-8 [89].

**Figure 1:**
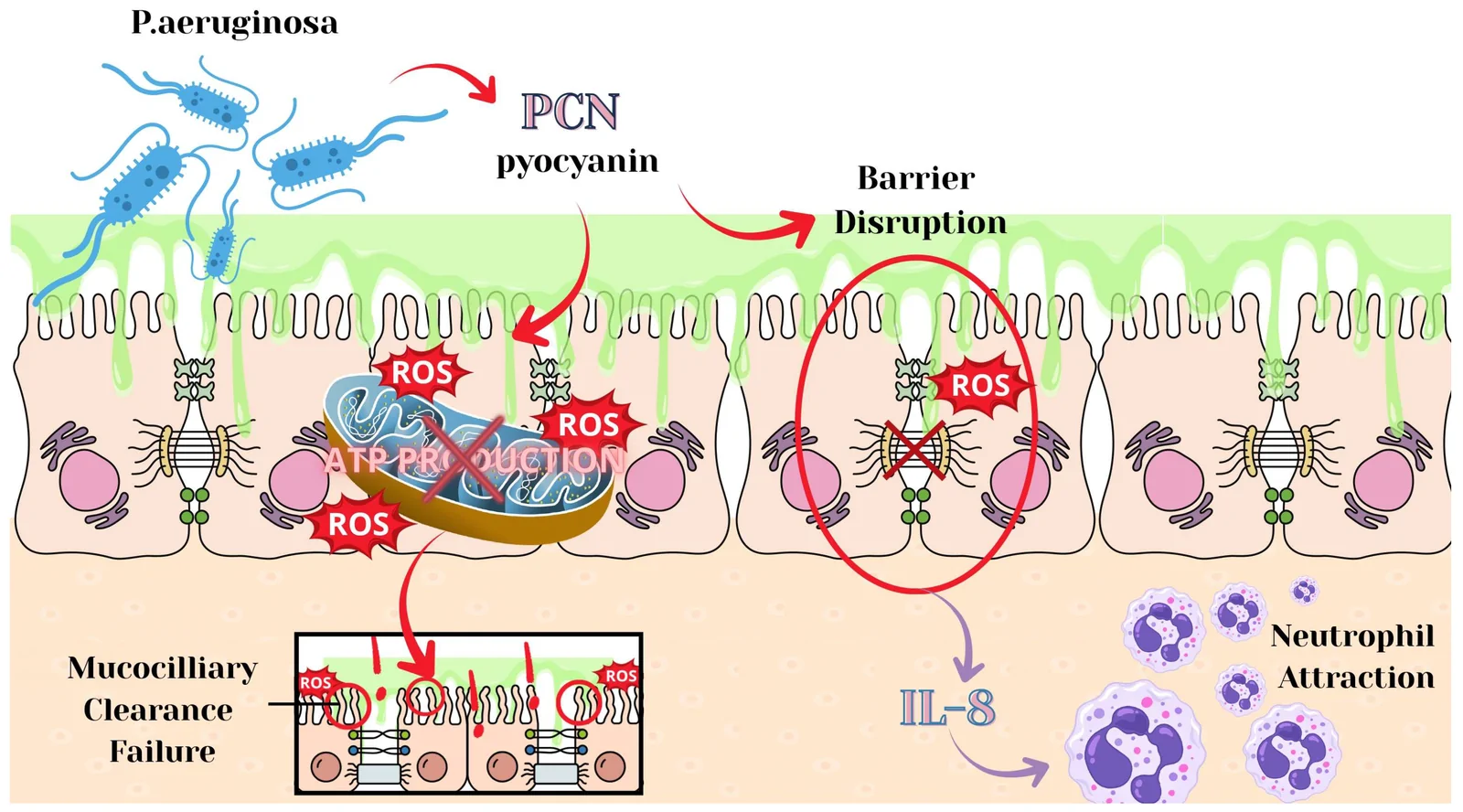
The role of *Pseudomonas aeruginosa* and its virulence factors in the pathogenesis of chronic sinusitis (CRS). Pyocyanin (PCN) produced by *P. aeruginosa* induces the overproduction of reactive oxygen species (ROS), leading to mitochondrial damage, ATP depletion, and impaired mucociliary clearance. Simultaneously, ROS and bacterial enzymes disrupt intercellular junctions, compromising the epithelial barrier and triggering IL-8 release, which attracts neutrophils to the site of inflammation. Red arrows indicate cause-and-effect relationships; purple arrows highlight cytokine release and immune cell recruitment; red “X” symbols mark inhibition or structural destruction (e.g., ATP synthesis deficit, cleavage of tight junctions); red circles highlight key pathological alterations in the course of CRS (epithelial barrier breakdown and ciliary dysfunction). Original figure created by the authors.

Taken together, these mechanisms illustrate the multifaceted role of *P. aeruginosa* in CRS pathogenesis, as summarized in Figure 1.

### 3.3. The Role of Staphylococcus aureus

Similarly to *P. aeruginosa*, *S. aureus* is found in healthy sinonasal samples but is significantly more prevalent in swabs from CRS patients [96,128]. With numerous virulence factors, it has been generally associated with the Th2 inflammation profile, found in a significant majority of CRSwNP patients [129,130]. *S. aureus* colonization prior to endoscopic sinus surgery was linked to a subjective negative outcome postoperatively [131].

*S. aureus* enterotoxin type B (SEB), a superantigen, has been linked to persistent inflammation and tissue damage and has been proposed as a potential risk factor of nasal polyposis [96]. Martens et al., based on an air–liquid interface culture, observed that upon SEB administration, nasal polyp epithelial cells from CRSwNP patients exhibited greater epithelial permeability. This was explained by SEB-induced activation of TLR2 on primary polyp and nasal epithelial cells, which led to increased IL-6 and IL-8 levels [89]. Yoon et al. carried out a study on primary human nasal epithelial cells (pHNECs) and reported that exposure to SEB causes overabundance of mitochondrial ROS and endoplasmic reticulum (ER) stress-induced inflammation, both of which may contribute to the clinical picture of CRSwNP [132,133]. Lipoteichoic acid (LTA) is another substance that can activate TLR2. Combined with CD14 receptor activation, LTA stimulates the release of pro-inflammatory cytokines and ROS [134], which may lead to increased epithelial permeability [135]. As a superantigen, SEB can activate T cells in a non-specific manner, promoting Th2 inflammation via IL-4, IL-5 and IL-13. These cytokines can, in turn, act on B cells to upregulate polyclonal IgE production, which bind to mast cells. Once activated, mast cells release numerous compounds, including strong eosinophil attractors [136]. Interestingly, Lan et al., using nasal tissues obtained from participants (patients with CRSwNP and a control group), found that even a non-enterotoxigenic *S. aureus* strain can stimulate nasal polyp tissue to produce Th2-associated cytokines, such as IL-5 and IL-13, along with IL-33 and long-form thymic stromal lymphopoietin (lfTSLP) [137]. Another study reported elevated IL-5 production in polyp tissues from CRSwNP patients infected by *S. aureus*, which visibly dropped after the bacteria were killed using a penicillin/streptomycin combination [138]. Murphy et al. [135] reported, based on an in vitro study on HNEC cultures in an air–liquid interface, that detrimental properties of *S. aureus* V8 protease on tight junctions via zona occludens-1 (ZO-1) protein decrease. A local decrease in IL-6 was also observed, which can be interpreted as a possible immune evasion mechanism of *S. aureus* [135]. Staphylococcal proteases, in general, are considered to slow down the epithelial repair rate and push the immune response towards Th2 [139,140]. Another virulence factor, alpha-hemolysin (Hla), can form transmembrane pores in eukaryotic cells and lead to impaired ciliary activity [141]. As iron is crucial to bacterial survival, *S. aureus* shows an impressive set of iron uptake and retention mechanisms, which becomes especially useful in an iron-scarce host environment [142,143]. *S. aureus* can also improve its survival chances by entering host cells [144]. In a study in which tissue samples were obtained from participants and immunohistochemistry was used for analysis, a higher prevalence of intracellular bacteria was associated with a greater risk of revision endoscopic surgeries [145]. Similarly to *P. aeruginosa*, *S. aureus* can take either planktonic or biofilm form. Fibronectin-binding proteins allow the staphylococci to adhere to the extracellular matrix, a critical step in biofilm creation [146]. The bacteria can also secrete Panton–Valentine leukocidins, strong NETosis inductors, in order to cause excess neutrophil death. Although NETs are bacteriocidal, *S. aureus* biofilms can use nucleases to break them down, which creates deoxyadenosine, a pro-apoptotic factor for macrophages [147,148]. *S. aureus* biofilms sampled from CRSwNP patients also have higher metabolic activity and positively correlate with eosinophil frequency, as well as disease severity, dominated by type-2 inflammation [149]. In an earlier study, swabs were collected from participants, and *S. aureus* was isolated through culture and species-level differentiation testing. After that, biofilms were formed in vitro. Based on that, Shaghayegh et al. positively correlated *S. aureus* biofilm occurrence with Th2-associated cells, whereas the correlation was negative for Th1 and Th17-associated cells [150]. It was also reported that CRSwNP patients increased antibody-secreting cells, mature naïve B cells, and class-switched memory B cells, as well as reduced Breg frequencies—all of which correlate with disease severity [151]. Panchatcharam et al. compared equal doses of planktonic and biofilm *S. aureus* exoproteins and reported that the latter exhibit significantly greater negative effects on the epithelial barrier, increasing its permeability by more than 250 times [152]. Somewhat conversely, some analyses have shown that, similarly to *P. aeruginosa*, *S. aureus* strains derived from chronic infections traded their virulence factors for increased persistence capabilities [153,154]. In order to adapt, bacteria reduce glycolytic activity and other costly biosynthesis pathways while upregulating salvage reactions, nutrient uptake and cofactor biosynthesis [153,155]. All these actions are presumably undertaken in order to adjust to the chronically inflamed mucosa of CRS.

**Figure 2:**
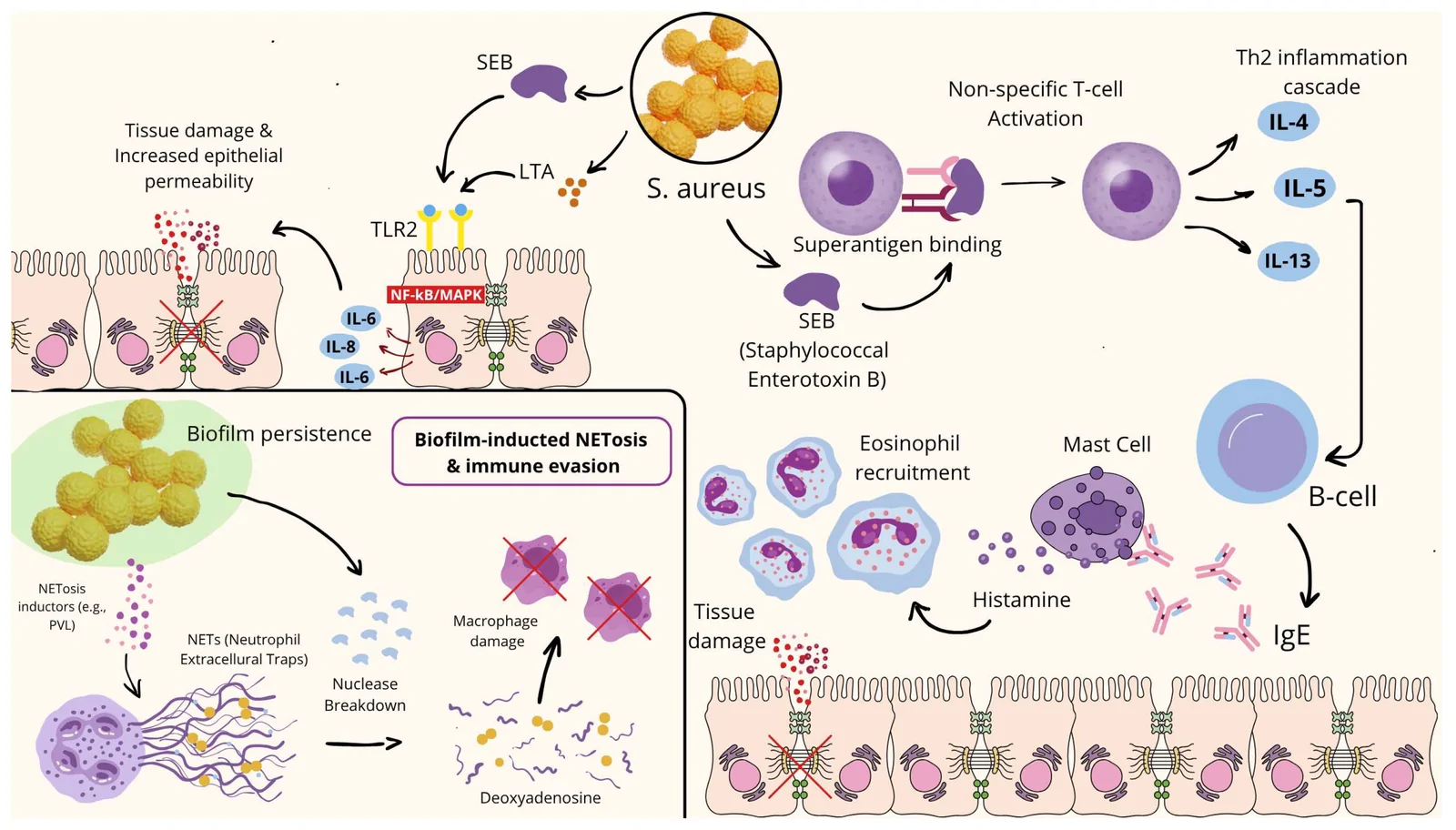
The role of *Staphylococcus aureus* and its virulence factors in the pathogenesis of chronic sinusitis with nasal polyps (CRSwP). Staphylococcal enterotoxin B (SEB) and lipoteichoic acid (LTA) activate TLR2/CD14 signaling, triggering NF-κB activation and driving pro-inflammatory cytokine secretion (IL-6, IL-8) by nasal epithelial cells. Concurrent non-specific T-cell activation by superantigens induces a robust Th2 immune profile (IL-4, IL-5, IL-13), leading to polyclonal IgE synthesis, mast cell degranulation, and massive eosinophil recruitment. These inflammatory cascades, together with staphylococcal proteases (e.g., V8 protease) and alpha-hemolysin (Hla)—which are not shown in the figure—severely disrupt epithelial tight junctions (e.g., ZO-1), increasing mucosal permeability and causing ciliary dysfunction. In the sub-panel (bottom left), staphylococcal biofilms induce neutrophil extracellular trap formation (NETosis) while using nucleases to degrade NETs into deoxyadenosine. As a result, both neutrophil death and macrophage apoptosis occur, which promotes evasion of the host immune response. Black arrows indicate cause-and-effect relationships; red “X” symbols denote structural disruption or cellular destruction (e.g., the cleavage of epithelial tight junctions and macrophage cell apoptosis). Original figure created by the authors.

The key virulence factors of *S. aureus* and their contribution to CRS pathogenesis are summarized in Figure 2.

### 3.4. From Virulence to Persistence: A Summary of Pathobionts’ Behaviours in the Course of CRS

The two most common pathobionts, *P. aeruginosa* and *S. aureus*, differ greatly in their suggested contribution to chronic inflammation at the center of CRS. Nevertheless, when the pro-inflammatory environment is set, both these bacteria seem to shift from presenting virulent traits to a persistence-oriented state [103,124,153,154]. *P. aeruginosa* as well as *S. aureus* can reduce biosynthesis pathways oriented at growth in order to focus on upkeeping biofilm and general pro-inflammatory processes, despite different means to do so [89,124,125,126,127,146,149,152] Considered together, these adaptations seem to resemble a shared survival strategy. This interesting convergence may further support the concept of CRS as an interconnected web of microbial persistence and multilayer host-related inflammatory processes.

### 3.5. Bacterial Biofilm as a Mechanism of Chronicity

A biofilm is a fixed group of homogeneous or heterogeneous bacterial cells surrounded by extracellular polymeric substances (EPSs) rich in proteins, polysaccharides, and eDNA [121,156,157]. The structure of a biofilm allows bacteria to avoid contact with host immune cells and competing microorganisms [158]. Furthermore, removing bacteria in a biofilm is significantly more difficult than eliminating planktonic microbiota [159], which plays a key role in the mechanism of chronic diseases. All of the above-mentioned biofilm characteristics constitute the foundation for bacterial survival, as the physical barrier of EPSs reduces the selective and metabolic pressures exerted on them (so-called persister cells), allowing for stable colonization of the sinuses. This, in turn, leads to a significant difference between the intensity of inflammation and its expected killing effectiveness, consequently promoting chronicity of the disease process on the one hand and deepening mucosal damage on the other [160]. Furthermore, biofilm appears on foreign bodies such as stents inserted into the sinus ostia to unblock these structures, particularly after Functional Endoscopic Sinus Surgery (FESS) [161]. For these reasons, interest in biofilm has increased significantly in recent years; the clinical and financial implications of its presence are being studied.

Besides reducing immune cell penetration, biofilm also reduces antibiotic penetration and increases bacterial tolerance. Antibiotic resistance is a multifactorial phenomenon. It has been proven that bacteria found in biofilm are up to 1000 times more resistant to antibiotic therapy than those not present [162].

We can distinguish several main groups of factors important in the pathogenesis of biofilm formation: physical, biochemical, environmental, and molecular factors [163,164]. Biofilm is fundamentally a physical barrier separating the bacterial environment from the outside world. This clinically unfavorable aspect is primarily due to the previously mentioned biofilm-forming extracellular polymeric substances (EPSs) [165], particularly their viscoelastic form [166].

The internal environment of a biofilm is heterogeneous, with structural layers differing in their living conditions. The more superficial the biofilm, the more oxygen is available; sometimes, in the zones closer to the center, oxygen is so low that it causes stress in bacterial cells. This results in the bacteria entering a metabolically dormant state, rendering these dormant cells significantly less sensitive to bactericidal substances [167]. It is worth mentioning that the inability of nutrients to reach the center of the biofilm will indirectly weaken the effect of bactericidal substances, affecting the cell’s metabolic cycle, as it will be partially or completely inhibited [168]. Therefore, it is crucial to select an antibiotic with a specific mechanism of action that will be effective against metabolically inactive cells or to use appropriate combination therapy [168,169]. Changes in environmental oxygenation result in local pH changes, which also contribute to non-uniform antibiotic penetration [167]. The biochemical factors influencing biofilm formation include Quorum Sensing (QS), which can be compared to a bacterial communication system. QS regulates the production of EPSs (by influencing genes), as well as virulence factors and the metabolic adaptation of bacterial cells. This type of communication is therefore crucial in the formation of biofilms and, consequently, antibiotic resistance [170]. Furthermore, signaling molecules used in QS are gaining increasing interest because they can detect the presence of biofilm at an early stage of diagnostic procedures [171], faster than with culture. When speaking about molecular factors, it is important to remember that the biofilm form provides greater opportunities for the transfer of resistance genes between cells, leading to mutations and ineffective antibiotic therapy [172]. Additionally, selecting an antibacterial drug from the wrong group or using the wrong dosage may lead to increased biofilm production. For example, ceftaroline at a concentration below the minimum inhibitory concentration (sub-MIC) leads to an increase in the *Staphylococcus aureus* biofilm mass [173].

The impact of biofilms on the immune system is also significant. Biofilm acts like a blanket, protecting the bacteria hidden beneath it from aggressive immune cells. This allows them to persist unnoticed in the human body and lead to chronic infections [158,174]. Some bacterial cells found in biofilms disrupt the balance of T lymphocytes, in favor of either Th1 or Th2. This often promotes host immune stagnation, which translates into the occurrence of chronic infection. This may occur, among others routes, by disrupting the phagocytosis of immune cells, e.g., by changing the phenotype of macrophages to the M2 type or by functional exhaustion of neutrophils [175,176]. Antibiotic resistance and its impact on the host immune system make biofilms a chronic problem in many diseases, such as CRS. The eradication of biofilms is difficult, which is why their presence is associated with disease recurrence [158,174]. Recurrence and lack of full recovery, however, result in enormous costs and an excessive burden on healthcare systems [177,178].

**Figure 3:**
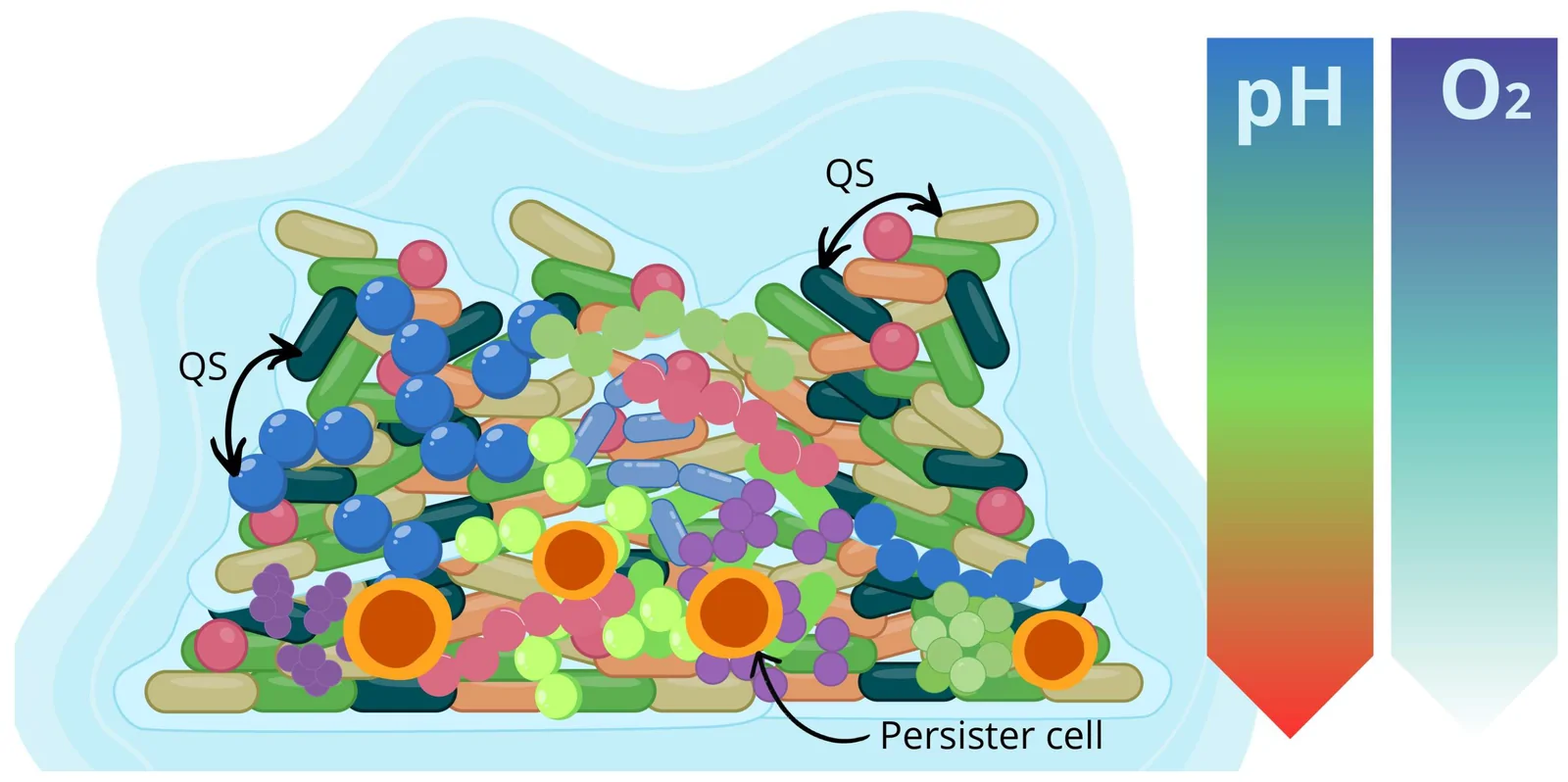
Structural organization and microenvironmental gradients within a bacterial biofilm. The graphic illustrates the complexity of biofilm structure as a multilayered structure comprising heterogeneous bacterial populations (represented by various shapes and colors) that creates favorable conditions for bacterial growth. A key mechanism that facilitates cooperation among these diverse cells in biofilm formation is cell-to-cell communication—quorum sensing (QS). The deeper into the biofilm structure, the poorer and more acidic the environment becomes in oxygen. These factors lead to the formation of persister cells, which are remarkably resistant to antibiotics. Original figure created by the authors.

The structural organization of biofilms and the associated microenvironmental gradients in oxygen availability and pH, together with quorum sensing and the presence of persister cells, are illustrated in Figure 3.

## 4. Factors Modulating the Nasal and Sinus Microbiome

### 4.1. The Influence of Gender on the Microbiome and the Course of Crs

Chiu et al. concluded that women are more likely to suffer from CRS than men, with CRSsNP significantly predominating, in contrast to men, who most commonly experience CRSwNP [179]. Furthermore, men have larger polyps in imaging studies than women [43]. CRSsNP predominates in every female age group, and after the age of 60, a general decline in the incidence of CRS, both CRSsNP and CRSwNP, is observed compared to men. This decline may be due to several factors: reduced mucus production, anatomical changes, or a weakening of the immune system that progresses with age [180,181,182,183]. Menopause could also be a possible explanation, but statistically, women go through menopause earlier than the age of 60–69 [179]. Some studies suggest that the higher incidence of CRS in women stems from the overlap of symptoms with co-occurring conditions such as depression or migraine. This phenomenon may contribute to the higher prevalence of CRS among women. However, this hypothesis remains unconfirmed, and it cannot be stated with certainty that the misdiagnosis of these conditions leads to an increase in CRS diagnoses in women [44,184]. Additionally, women are more susceptible to the effects of tobacco and hormones, which may also lead to more frequent sinus disease [44]. Women suffering from CRS have more severe symptoms and are more likely to develop depression during sinus disease [43]. The differences in the incidence of CRS types can also be traced to the sinus microbiome. The nasal microbiome in men is characterized by a higher bacterial density and pathogen colonization compared to women. Moreover, after culture of nasal swabs on a blood agar and on a medium selective for *S. aureus*, Stensen et al. demonstrated that male gender is a risk factor for more frequent colonization and infection with *Staphylococcus aureus* [185], the presence of which in the upper respiratory tract may intensify the eosinophilic state, predisposing to CRSwNP [186], which would explain the higher incidence of this type of CRS in men. On the other hand, the female microbiome is characterized by more stable nasal communities, dominated by commensals [58].

### 4.2. The Impact of Sex Hormones (Estrogens) on the Nasal Microbiome

There is a growing body of evidence suggesting that sex hormones may influence the function of the upper respiratory tract mucosa and modulate the immune response within the paranasal sinuses [45,46]. Estrogens and progesterone act on immune cells, including lymphocytes, macrophages, and dendritic cells, regulating the production of cytokines and inflammatory mediators. Estrogens have been shown to influence the Th1/Th2 immune response balance and modulate inflammatory processes within the mucosa [45,46]. Estradiol, used in hormonal contraception, helps delay the apoptosis of neutrophils, which predominate in a Th1-biased immune response; a Th1-biased immune response is characteristic of CRSsNP. When a patient reaches menopause, her estrogen levels decrease, which causes a decrease in neutrophils, which should exacerbate CRSsNP. However, this hypothesis has not been proven, and further studies examining this relationship in depth are needed. Additionally, estradiol helps alleviate CRSsNP by inhibiting TNF-α, NK cells, and IFN-γ, which have proinflammatory properties; the main inflammatory mediators in CRSsNP are IFN-γ and TGF-β [45,46]. Although estrogens primarily influence the Th1/Th2 balance, as mentioned above, growing evidence indicates that they can also affect the Th17-type response in conditions such as bronchial asthma and vaginal candidiasis [187,188]. Importantly, some of our considerations regarding the role of sex hormones are derived from experimental studies conducted in murine models or in patients with other inflammatory and dysbiosis-associated diseases, rather than in patients with CRS. While such studies provide valuable mechanistic insights, their findings cannot be directly extrapolated to the CRS population. Thus, the proposed associations should currently be regarded as hypothetical and require validation in well-designed studies involving patients with CRS. Further research specifically addressing sex hormone-related mechanisms in CRS is therefore warranted to determine whether observations from experimental models and other disease contexts can be translated to CRS [187,188]. Experimental studies by Fuseini et al. (2019) in mouse-derived Th17 cells indicate that ERα signaling promotes IL-17A production, whereas ERα deficiency reduces IL-17A production, partly through effects on IL-23R expression and Th17 cells [187]. In CRS, IL-17A, a major cytokine produced by Th17 cells, is a potent chemotactic factor for neutrophils and plays a significant role in the pathogenesis of neutrophilic rhinosinusitis type 3 [46,189]. However, these observations do not establish a direct causal link between estrogen signaling, Th17/IL-17A activity, and type 3 inflammation in CRS. Rather, the available evidence suggests a potential association that warrants further investigation. In particular, it remains unclear whether alterations in estrogen signaling contribute to CRS-associated inflammation, arise as a consequence of the inflammatory process, or interact with other factors such as microbial dysbiosis. Analysis of estrogen characteristics led to the conclusion that ECC (estrogen containing contraception) reduces the risk of developing CRSsNP, while no correlation was observed in the case of CRSwNP [190]. *Corynebacterium propinquum*, which is part of the physiological microbiome of the nose and sinuses (detected using 16s rRNA sequencing) has been proven to inhibit the growth of potentially pathogenic bacteria and maintain the balance of bacterial cell communities (more in Section 2), exhibiting an inverse relationship with Th1 and Th2 [73,191]. This raises hopes for the use of estrogen in CRS treatment, as estradiol causes a decrease in cytokines such as IFN-γ, which leads to stabilization of the *C. propinquum* population. Considering the protective role of this species in inhibiting the expansion of pathobionts, hormonal fluctuations (e.g., during menopause) may be a significant risk factor for the loss of microbiome homeostasis and the transition to a state of chronic dysbiosis. However, in order to substantiate our assumptions that the body’s hormonal status can influence the interplay between the inflammatory signaling pathway and the sinonasal microbiome—and to implement estrogen therapy for sinonasal diseases—we must validate this through extensive scientific research, as this relationship has not previously been studied in the context of CRS. Another important element of the action of sex hormones is their influence on the integrity of the epithelial barrier. Estrogen receptors are present in respiratory epithelial cells, and their activation can influence the expression of intercellular junction proteins and the regulation of mucus production. This occurs, among other things, by modulating the dynamics of aquaporin 5 (AQP 5), which, by regulating the integrity and stability of tight junctions, can lead to swelling and polyp formation in CRS. A downward trend in AQP 5 expression was observed in CRSwNP (although the difference was not statistically significant), which may indirectly result in fluid retention in the intercellular space and, consequently, polyp formation. Immunohistochemical studies observed a decrease in AQP 5 expression in the mucosa of nasal polyps through reduced expression on the cell surface. Additionally, it has been noted that AQP 5 expression increases during the follicular phase of the menstrual cycle (during which estrogen levels are high) [192], which further emphasizes the significant role of this hormone in the potential treatment of CRS. However, further studies are needed to consider the role of aquaporin 5 in the pathomechanism of sinus diseases. Consequently, hormonal changes may modify the local microbial environment by affecting host defense mechanisms, including mucociliary clearance and mucosal barrier function [46].

Although direct studies examining the effects of sex hormones on the sinus microbiome are currently limited, epidemiological data suggest a potential link between hormonal status and upper respiratory tract diseases. Systematic reviews of nasal and sinus diseases have highlighted that changes in estrogen and progesterone levels may influence inflammatory symptoms and the course of diseases such as allergic rhinitis and chronic rhinosinusitis [43,44].

Therefore, it is hypothesized that hormonal fluctuations may indirectly influence the stability of the sinus microbiome by modulating the immune response and mucosal barrier properties [184,193]. However, these mechanisms remain poorly understood and require further investigation, particularly using modern microbiome analysis methods.

### 4.3. Impact of Environmental Pollutants on the Microbiome and the Mucosal Barrier

#### 4.3.1. Air Pollution

A person inhales approximately 11,000 L of air daily, so air quality is crucial to human health [47]. Air pollution has become a serious international health and ecological problem in recent decades. It is estimated that 6.7 million people die prematurely each year due to air pollution [194]. A growing body of research is being conducted on smog and its impact on human health. Air pollution affects the human respiratory tract and its microbiome. Air pollution affects the human body by stimulating several pathways: the Toll-Like Receptor (TLR) pathway, the reactive oxygen species (ROS) sensing pathway, and the carbohydrate sensing pathway [47]. TLRs found on epithelial cells, including those in the nasal cavity [195], are responsible for detecting pathogen-associated molecular patterns (PAMPs), and their role is crucial in initiating the immune response. Particulate matter (PM) most often binds to TLR-4 or TLR-2 [47]; TLR-4 is a receptor that detects both inflammatory and non-inflammatory signals, such as those related to ischemia [195]. PM, as an irritant present in polluted air, will exacerbate the proinflammatory effect of TLR activation, which may lead to dysbiosis [195].

PM activates NADPH oxidase and leads to mitochondrial dysfunction [196]. These factors increase ROS levels, resulting in local inflammation and immune activation. Increased ROS leads to a local decrease in pH, which disrupts the growth of acid-sensitive commensals (*Firmicutes* and *Bacteroides*) [197], which may contribute to dysbiosis. Additionally, ROS damage epithelial cells and influence various interactions between bacteria, leading to chronic inflammation. Furthermore, bacterial cells living in the nasal cavity most likely metabolize air pollutants, which can influence their living environment and also may lead to dysbiosis. In the 2020 study by Xue et al., the gut-pulmonary axis was further proposed as a mechanism contributing to dysbiosis [197].

PM and cigarette smoke increase epithelial permeability by degrading tight junctions, which can lead to exacerbated inflammation, immune imbalance, and the entry of bacterial toxins, LPS, or contaminants into the bloodstream. Furthermore, it should not be overlooked that a healthy epithelium secretes antimicrobial peptides and IgA antibodies, and PM can also alter antibody secretion, which is crucial in repelling potentially dangerous microorganisms. Cigarette smoke can enhance bacterial adhesion by inducing the production of a specific protein, FimA, and increase the diversity of the upper respiratory tract (URT) community. All of this increases the risk of *S. aureus* invasion and biofilm formation [198]. In China, a study was conducted in 2025 comparing the nasal microbiome of healthy individuals briefly exposed (21 days) to traffic-related air pollution (TRAP) with those who were not, using 16S rRNA sequencing. It turned out that individuals exposed to TRAP had increased microbial diversity in the nasal cavity compared to unexposed individuals. Samples from individuals exposed to TRAP showed an increase in bacteria from the *Firmicutes* (the largest changes), *Bacteroidota*, and *Actinobacteria* phyla. Within the phylum *Firmicutes*, changes occurred in *Clostridiacae, Peprostreptococcales, Lactobacillaceae*, and *Lachnospiraceae* [199]. The increased prevalence of *Lactobacillaceae* and *Lachnospiraceae,* which are defined as beneficial bacteria, and the parallel increase in potentially pathogenic bacteria indicate that the nasal microbiome is characterized by complex processes, multiple interactions, and self-regulation. The study concluded that even short-term exposure to air pollution disrupts the nasal microbiome and increases its diversity [199].

#### 4.3.2. Ozone, Humidity, and the Gut–Lung Axis

In 2020, Niu et al. [200] analyzed the microbiome of people who were exposed to ozone pollution using 16S rRNA sequencing. The study revealed that ozone, as a strong oxidant, reduces the diversity of the microbiota and the number of nasal commensals, *Actinobacteria* and *Firmicutes*, which may protect against infections. However, it increases the number of potentially pathogenic *Moraxellaceae* and *Pseudomonadaceae*. Therefore, even short-term exposure to ozone can lead to the disruption of the nasal microbiome and increased susceptibility to upper respiratory tract infections [200]. In addition to air pollution, meteorological and seasonal factors can influence the composition of the nasal microbiome. A long-term multiomic study by Sailani et al. [201] including 105 individuals from the San Francisco Bay Area followed for up to four years demonstrated significant seasonal variation in the nasal and gut microbiome, with changes being more dynamic within the nasal microbiome. Lower diversity of sinus microorganisms was observed in spring (coinciding with the regional allergy season), and several taxa associated with upper respiratory tract homeostasis, including *Staphylococcus*, *Porphyromonas*, *Dolosigranulum*, and *Corynebacterium*, exhibited distinctive seasonal patterns. *S. aureus* abundance increased in September and October [201]. The identified biological patterns were associated with temperature, solar radiation, atmospheric pressure, humidity, precipitation, and exposure to pollen and fungi. This suggests that seasonal variation in the nasal microbiome may result from the overlapping influence of multiple environmental factors rather than a single parameter [201]. More direct evidence linking environmental conditions to the nasal microbiome was provided by Perez-Garcia et al. [202], who analyzed nasal swabs from 416 adolescents (average age of approximately 13 years). Using 16S rRNA sequencing, the authors examined the associations between the nasal microbiome and short- and long-term exposure to PM_25_, NO_2_, O_3_, temperature, relative humidity, and residential greenery [202]. Short-term exposure to NO_2_ was associated with higher bacterial richness and diversity, while higher temperature was associated with lower diversity. Furthermore, the *Moraxella*-dominant nasal microbiome was associated with lower ozone exposure and higher temperature and relative humidity in the previous week. The abundance of selected bacterial genera, including *Staphylococcus* and *Corynebacterium*, was also associated with exposure to environmental factors and respiratory health parameters [202]. Because this study was observational, these associations do not allow for the determination of a cause-and-effect relationship; however, they indicate that environmental conditions may be a significant factor shaping the nasal microbiome.

A potential mechanism mediating these associations is the gut–lung axis. Disturbances in the gut microbiome (e.g., due to inhalation of contaminants from water or food) can affect the production of metabolites such as short-chain fatty acids (SCFAs) and immune signaling involving bacterial products, including lipopolysaccharide (LPS). These changes may modulate the systemic immune response of the mucosa and consequently influence the microbial environment of the respiratory tract [197].

The main environmental factors associated with alterations in the nasal microbiome and mucosal barrier, together with their potential pathophysiological consequences, are summarized in Table 2.

#### 4.3.3. Nose–Brain Axis

The importance of the nose–brain axis has been overlooked and downplayed for years, but increased research is currently underway. Researchers have begun to analyze the condition of the nasal cavity in patients with neurodegenerative diseases and concluded that the strong correlations between them cannot be coincidental. Studies suggest that exposure to allergens, infections, or toxins may alter the microbiome in a way that potentially promotes dysbiosis and local inflammation [203]. This, in turn, damages nerve axons and can result in the activation of central microglia, astrocytes, and impaired olfactory bulb function [204]. However, pathological changes do not have to stop at the bulbar level but can spread further throughout the central nervous system, causing neurological symptoms as well as anxiety, depression, and migraine headaches [205,206]. Inflammation-induced damage to the bulbar and microglia, along with psychological disturbances, are consistent with the clinical picture of patients with CRS. This suggests that the olfactory disorders observed in these patients may not only result from excess mucus and the presence of polyps but also from a mechanism involving the central nervous system.

#### 4.3.4. Chronic Stress and the Microbiome

Chronic stress activates the hypothalamic–pituitary–adrenal (HPA) axis, resulting in sustained glucocorticoid release. Glucocorticoids exert complex immunomodulatory effects, including suppression of cellular immunity and altered cytokine profiles [207,208]. Experimental models have shown that stress-induced HPA activation can alter the structure of the microbial community, particularly within the gastrointestinal tract [209,210]. Although direct evidence on the sinonasal microbiome is limited, mucosal immune modulation and barrier alterations induced by chronic stress may theoretically contribute to microbial instability in chronic rhinosinusitis. Clinically, cohort studies have shown that chronic rhinosinusitis is associated with an increased risk of anxiety and depressive disorders [211,212], suggesting that the nose–brain axis does not only operate unilaterally and potentially shows a bidirectional interaction between psychological stress and inflammation of the paranasal sinuses.

It is important to remember that the nose–brain axis has enormous therapeutic potential. Administering medications for neurodegenerative diseases (as well as other conditions) intranasally, rather than intravenously or orally, increases drug bioavailability because it bypasses the blood–brain barrier [204,213]. This form of administration is also less inconvenient for the patient than taking medications intravenously in a hospital setting. However, the nose–brain axis, as a therapeutic route, requires much more research to confirm its potential properties.

## 5. Discussion

Chronic rhinosinusitis (CRS) is now increasingly being viewed not as a chronic infection caused by a single pathogen but as a complex disruption of the homeostasis between the microbiome, mucosal epithelium, and the host immune response. Available data indicate that CRS is associated with reduced microbiome stability and diversity, as well as changes in the proportion of commensal and potentially pathogenic microorganisms [37,39,97]. Reductions in the abundance of *Corynebacterium* and *Dolosigranulum* have been reported in particular, while a higher proportion of *Staphylococcus*, including *S. aureus*, has been observed in CRSwNP [52,54,98,99]. At the same time, a single, universal definition of sinus dysbiosis has not yet been established [37,39].

Importantly, the relationship between dysbiosis and inflammation likely has a bidirectional nature. Microbiome disruption may contribute to damage of the epithelial barrier and activation of the inflammatory response, while chronic inflammation, impaired mucociliary clearance, and altered mucus properties may in turn contribute to the persistence of an abnormal microbiome [96]. Thus, these interrelated processes may form a self-sustaining vicious cycle in which dysbiosis, mucosal barrier disruption, and abnormal immune response mutually reinforce each other.

Biofilm may play a significant role in maintaining this condition. In the study by Danielsen et al. (2015), biofilm was found in 81.5% of patients with CRSsNP and 97.1% of patients with CRSwNP [214]. Bacteria in biofilms are protected by the extracellular matrix, have altered metabolism, and exhibit greater tolerance to antibiotics [158,174]. Consequently, bacterial eradication is difficult, and the presence of biofilm is associated with disease relapse [158,174]. This has direct implications for the contemporary treatment of CRS. Antibiotic therapy remains an essential element of treatment in selected patients, but it does not always lead to the restoration of a normal microbiome. Kim et al. (2021) demonstrated that antibiotic use reduces the differences between the microbiomes of healthy individuals and patients with CRS, leading to the loss of some commensals and an increased similarity to the profile observed in CRS [95,215]. Therefore, microbiome-targeted therapy could play an important role in the future management of CRS, aiming to restore the microbial balance in the sinuses disturbed by CRS. It is important to note that we strongly believe that such treatment should not be considered a replacement for standard therapy at this time but a potential complement to anti-inflammatory therapy, antibiotics, steroids, and FESS [95].

One of the most easily available methods for this is probiotics. Experimental studies have shown that selected *Lactobacillus* strains can influence epithelial integrity and the immune response; for example, *L. casei* affects TLR2 and can improve the organization of epithelial tight junctions in models based on CRSwNP material [89]. Currently available reviews describe probiotic therapy as an adjunct therapy for CRS, available in both oral and intranasal forms, with the potential goal of increasing the diversity of protective microorganisms and reducing disease symptoms. However, it should be emphasized that probiotic effects are strain-dependent, so results obtained for one species cannot be automatically transferred to others [89].

An even more direct strategy for restoring the disturbed microbiome is sinonasal microbiota transplantation (SNMT), which involves the transfer of microorganisms from a healthy donor to the nasal cavity and sinuses. This method, unlike strategies targeting single strains, involves the transfer of the entire microbial community, thereby attempting to recreate a functional ecosystem [216]. In a pilot study by Mårtensson et al., 22 patients with CRSsNP were first treated with a 13-day course of antibiotics, followed by a five-day course of SNMT, resulting in a significant improvement in SNOT-22 scores and increased microbiota diversity lasting up to 3 months [217]. However, this method remains in the early stages of development and requires randomized trials and standardization of, among other things, the method of administration, dose, and donor qualification [216,218]. Therefore, SNMT should currently be considered a prospective adjunct to, rather than an alternative to, standard CRS treatment.

Another potential direction is bacteriophage therapy, particularly in the case of biofilm and multidrug-resistant bacteria. Fong et al. demonstrated, in vitro, that a cocktail of four bacteriophages reduced the biomass of *P. aeruginosa* biofilms from CRS patients, with a maximum reduction of 99.6% after 24 h [219]. Bacteriophages can also be combined with antibiotics, as the enzymes they produce, including depolymerases, can destabilize the biofilm matrix and increase the bacteria’s accessibility to antibiotics [220,221]. However, this method is currently in preclinical and clinical development, without standardized treatment protocols [222,223].

The use of nanotechnology should be assessed similarly. Nanoparticles can improve penetration through mucus and biofilm, have direct antibacterial effects, or serve as antibiotic carriers [224,225]. In vivo studies have demonstrated the antibiofilm effect of silver nanoparticles, but further research on the safety and toxicity of these compounds is necessary [226].

Diagnostics based on microbiome characterization may also be an important area of development. Classic microbiological methods do not provide a complete picture of the sinus ecosystem; therefore, next-generation sequencing (NGS), including metagenomic next-generation sequencing NGS (mNGS), may be particularly important. Alhtough some centers offer microbiome analysis using mNGS, standardization of sample collection, sequencing, and result interpretation procedures is necessary before routine use [63].

In the future, microbiological diagnostics may be combined with inflammatory endotype assessment. In addition to mNGS, biomarkers such as periostin, eotaxin-3, IL-5, and eosinophil cationic protein are being studied [227,228,229]. Methods utilizing artificial intelligence and radiomics are also being developed, which may aid in endotype recognition and recurrence prediction [230,231]. This combination of microbiological, immunological, and imaging data may enable more precise therapy selection in the future.

The potential influence of sex hormones is also noteworthy. Estrogens may influence the immune response, epithelial barrier integrity, and mucociliary clearance mechanisms, in addition to indirectly affecting the microbial environment of the sinuses [43,44,46]. Estrogen-containing contraceptive use was shown to be associated with a lower risk of CRSsNP, while no such association was observed for CRSwNP [190]. Available data on hormone replacement therapy suggest a possible reduction in the need for ESS but also indicate a potential shift in the inflammatory profile toward a more neutrophilic inflammatory response and a greater need for antibiotics [184]. For this reason, HRT currently remains a hypothetical treatment option, not a proven treatment for CRS.

Table 3 was created to summarize the emerging therapeutic approaches described above that target the sinonasal microbiome as well as their microbiota-mediated mechanisms, current evidence levels, major limitations, and general safety profile.

The importance of this research is heightened by the fact that CRS poses a significant burden on both patients and the healthcare system. The disease impacts quality of life and generates direct costs, including those related to consultations, diagnostics, pharmacotherapy, and procedures, as well as indirect costs resulting from, among other things, reduced productivity and limitations in daily functioning [1,2]. Relapses and the need for retreatment are a particularly significant problem; their frequency and difficulties in biofilm eradication increase the burden on the healthcare system [177,178]. For example, in the United States alone, the direct costs of treating CRS ranged from $10 to $13 billion in 2017, while indirect costs resulting from presenteeism and work abstinence reached $20 billion [233].

### 5.1. Limitations

The most significant limitation of this review is the significant heterogeneity of the available studies. Differences include populations, CRS phenotypes and endotypes, sampling location and method, microbiological methods used, and criteria for defining a healthy microbiome. The lack of a unified definition of sinus dysbiosis hinders direct comparison of results [37,39].

A second limitation is the inability to clearly determine whether dysbiosis is the cause or consequence of chronic inflammation. This relationship is likely bidirectional, but most available studies are observational and do not allow for the establishment of a cause-and-effect relationship. Furthermore, some potential therapies, particularly bacteriophages, nanotechnology, and HRT, are still in the preclinical stage or require larger clinical trials [184,223].

Another limitation is the predominance of data on bacteria in the current literature and, consequently, in this review. Although fungi and viruses are recognized components of the paranasal sinus ecosystem [36,49,64,65,66,67,68], evidence regarding their quantitative and functional changes during chronic rhinosinusitis is far less extensive than that concerning bacterial communities. Therefore, the bacteria-focused perspective presented in this review does not fully reflect the potential contribution of the paranasal sinus mycobiome and virome to the dysbiosis associated with chronic rhinosinusitis. Further research integrating the bacterial, fungal, and viral components of the paranasal sinus microbiome is needed to better characterize their interactions and potential role in disease persistence.

A significant aspect to consider in the management of CRS is the impact of treatment on the microbiome’s composition and dynamics. Commonly used antibiotics and corticosteroids affect various microbiota structures and specific taxa [26,27,28,95]. The direct bactericidal and bacteriostatic actions of antibiotics play a clearly significant role in shaping the microbiological profile [26,27,28]. In contrast, corticosteroids exert an indirect effect: they alter the sinonasal microbiome environment by modulating the host’s local immune response, which subsequently influences microbiome composition [26,27,28]. The impact of antibiotic and steroid therapies on the sinonasal microbiome is heterogeneous. Literature describing the relationship between treatment and microbiota species composition reveals considerable variability [26,27,28]. Therefore, attributing observed microbiological differences solely to the pathophysiological process of CRS requires accounting for the confounding effects of the administered treatments.

### 5.2. Future Perspectives

The most important direction for future research should be to further define standardized, functional characteristics of the sinus microbiome and more complex relationships between specific species rather than simply determining the presence of individual bacteria. The use of mNGS is particularly promising, as it can provide more comprehensive information on the composition and functional potential of microbial communities [63].

In parallel, longitudinal studies are needed to determine whether specific microbiome changes precede the development of CRS or only appear following chronic inflammation and treatment. It will also be important to develop methods for predicting response to antibiotics, steroids, biological anti-inflammatory treatments, and FESS based on the combination of microbial profile and immune endotype.

In terms of treatment, the most rational approach currently seems to be combining standard CRS treatment with attempts to restore microbial homeostasis, rather than replacing existing methods. Probiotics may offer a relatively clinically relevant adjunctive approach, while bacteriophages, advanced nanotechnology systems, and hormonal modulation require further investigation [89,184,219,223]. It therefore appears that the future of CRS treatment may lie not in the complete elimination of microorganisms but in controlling inflammation, limiting pathogens, and simultaneously restoring a stable microbiome and mucosal barrier integrity. Such an approach may ultimately reduce relapses, reduce unnecessary antibiotic exposure, and consequently reduce both the direct and indirect burden of CRS on patients and the healthcare system.

## 6. Conclusions

Chronic rhinosinusitis should increasingly be viewed not as a disease caused by a single pathogen but as a disruption of a complex ecosystem in which the sinus microbiome, epithelial barrier, and host immune response remain in a state of dynamic equilibrium. Available data suggest that disruption of this equilibrium may contribute to a self-reinforcing process in which dysbiosis, mucosal barrier dysfunction, and chronic inflammation mutually influence one another.

This approach also changes the way we interpret the presence of potential pathogens. *Staphylococcus aureus*, *Pseudomonas aeruginosa*, and biofilm-forming bacteria should not be considered solely as independent eradication targets but as components of an altered microbiological environment. At the same time, the heterogeneity of CRS and the lack of a clear definition of dysbiosis demonstrate the need for further elucidation of the causal relationships between the microbiome and chronic inflammation.

In our opinion, the consequence of this paradigm should be a gradual shift in both the diagnosis and treatment of CRS. The development of methods for assessing the functional state of the microbiome, combined with characterization of the inflammatory endotype and epithelial barrier status, may, in the future, enable more precise determination of disease mechanisms in individual patients. Simultaneously, evolving microbiome-targeted strategies—from modulation of selected microorganisms to more comprehensive attempts at ecosystem restoration—open the possibility of moving away from treatments focused solely on suppressing the effects of the disease.

The future of CRS treatment may therefore lie not only in more effective bacterial elimination or inhibition of inflammation but also in restoring conditions under which a normal sinonasal ecosystem is once again capable of maintaining homeostasis. In this sense, the concept of CRS as chronic dysbiosis may provide not only a new perspective on the pathophysiology of the disease but also a starting point for the development of the next generation of diagnostics and therapies.

## Figures and Tables

**Table 1:** Characteristics of type 1, type 2, and type 3 chronic rhinosinusitis according to inflammatory profile.

Feature	Type 1 (Th1-Dependent)	Type 2 (Th2-Dependent)	Type 3 (Th17-Dependent)	References
Main effector cells	Th1 cells	Th2 cells, eosinophils	Th17 cells, neutrophils, macrophages	[11,13]
Key cytokines	IFN-γ, IL-12, IL-2, TNF-β	IL-2, IL-4, IL-5, IL-13	IL-17, IL-22, IL-23	[8,9]
Defense mechanism	Against intracellular pathogens, induction of cytotoxic and local inflammation	Against parasites and allergens	Against extracellular pathogens (bacteria, fungi)	[9]
Inflammation characteristics	Cytotoxic, local	Eosinophilic, high IgE levels	Neutrophilic, often purulent	[8,9]
Most common phenotype	More commonly CRSsNP	Strong correlation with CRSwNP	Heterogeneous	[4,5]
Clinical associations	Lower tendency for polyps	Asthma, polyps, anosmia, allergic mucin	Purulent discharge, tissue fibrosis, edema	[4,11]
Geographic distribution ^a^	Global, coexists with Type 3 in the East	Dominant in Western countries; increasing in Asia	Dominant in Asia (Eastern countries)	[4,12]
Treatment response	Tendency towards resistance, weaker GCS response	Good response to GCS; high recurrence after FESS	Tendency towards resistance, weaker GCS response	[11,13]

^a^ The geographic distribution of CRS endotypes is not static and is subject to epidemiological changes. Although type 1 and type 3 inflammatory profiles were historically observed more frequently in Asian populations, recent years have seen an increase in the prevalence of CRSwNP characterized by a dominant type 2 inflammatory response [4,12]. **Abbreviations**: Th1—T helper 1; Th2—T helper 2; Th17—T helper 17; IFN-γ—interferon gamma; IL—interleukin; TNF-β—tumor necrosis factor beta; IgE—immunoglobulin E; CRSwNP—chronic rhinosinusitis with nasal polyps; CRSsNP—chronic rhinosinusitis without (sans) nasal polyps; GCS—glucocorticosteroid; FESS—functional endoscopic sinus surgery.

**Table 2:** Impact of environmental factors on the nasal microbiome and mucosal barrier.

Environmental Factor	Change in the Microbiome	Pathophysiological Outcome	References
Particulate matter (PM)	⭣ *Firmicutes*, ⭣ *Bacteroidetes*	TLR4 activation, pH decrease, ROS	[47,197,202]
Tobacco smoke	*S. aureus* (FimA protein) adhesion	Epithelial barrier damage, biofilm	[198]
Ozone (O_3_)	⭣ Diversity ⭣ *Actinobacteria, Firmitues*, ⭡ Moraxellaceae, *Pseudomonadaceae*	Significant cellular oxidative stress, risk of upper respiratory tract infection	[200,202]
Smog (TRAP)	⭡ Diversity (brief)	Unstable community autoregulation	[199,202]
Nitrogen dioxide (NO_2_)	⭡ Bacterial richness and diversity (short-term exposure)	Modulation of nasal microbiome composition	[201,202]
Higher humidity (relative and environmental)	Association with *Moraxella*-dominant microbiome; contributes to seasonal microbiome variation	Weakening of the physical barrier (seasonal), modulation of respiratory homeostasis	[201,202]
Higher temperature	⭣ Diversity; distinct seasonal patterns affecting taxa (e.g., *Staphylococcus*, *Porphyromonas*, *Dolosigranulum*, and *Corynebacterium*)	Environmental variation and overlapping influence on respiratory homeostasis	[201,202]

**Abbreviations**: PM—particulate matter; TLR—Toll-Like Receptor; ROS—reactive oxygen species; TRAP—traffic-related air pollution; O_3_—ozone; FimA—fimbrial adhesin protein. ↓—decrease; ↑—increase.

**Table 3:** Emerging microbiome-targeted therapeutic approaches for chronic rhinosinusitis: mechanisms, evidence level, limitations, and safety profile.

Therapeutic Approach	Microbiota-Mediated Mechanism	Evidence Level	Main Limitations	Safety Considerations	References
Probiotics	Competitive exclusion of pathogens through the production of metabolites (e.g., lactic acid) altering the microenvironment. Enhancement of epithelial integrity by interaction with specific cell receptors	Ex vivo (human nasal mucosal biopsies), in vivo (mouse model) and in vitro models	Absence of in vivo human studies; agar-based assays limit the diffusion of macromolecular compounds, which hinders the detection of antimicrobial agents other than lactic acid	Generally considered favorable (*Lactobacillus* strains constitute the natural microbiota of the upper respiratory tract); clinical safety data remain limited in CRS	[89,232]
Sinonasal microbiota transplantation (SNMT)	Re-establishment of healthy microbiota using donor transplantation	Open-label, randomized case series	Small sample size; early-stage development; requires further research. The requirement for antibiotic administration in some patients disrupted microbiota restoration and SNOT-22 results	No adverse events reported	[216,217,218]
Bacteriophage therapy	Anti-biofilm and bactericidal activity associated with the infection of target bacteria, intracellular replication, and lysis of bacterial host	In vitro study (on ex vivo *P. aeruginosa* clinical isolates)	Absence of in vivo human studies. Unpredictable, non-linear dose-dependent effect due to phage self-replication	Not fully established; requires further clinical trials	[219,220,221]
Nanotechnology-based approaches	Antibacterial effect associated with impairment of bacterial metabolism, protein synthesis and induction of oxidative stress in bacteria	In vitro studies (clinical isolates from patients with CRS); in vivo study (rabbit model of sinusitis)	Absence of in vivo human studies. The synergistic effects with antibiotics remain unpredictable and unclear	Not fully established; requires further research on toxicity of nanoparticles	[224,225,226]

**Abbreviations**: SNMT-sinonasal microbiota transplantation; SNOT-22-22-item Sino-Nasal Outcome Test.

## Data Availability

No new data were created or analyzed in this study. Data sharing is not applicable to this article.

## Abbreviations

The following abbreviations are used in this manuscript:

CRS	Chronic rhinosinusitis
CRSwNP	Chronic Rhinosinusitis with Nasal Polyps
CRSsNP	Chronic Rhinosinusitis without (sans) Nasal Polyps
EBV	Epstein–Barr virus
HHV-6	Human herpes virus-6
agr	Accessory gene regulator
CAMP factor	Christie–Atkins–Munch–Petersen factor
RSV	Respiratory syncytial virus
TLR	Toll-Like Receptor
lfTSLP	Long-form thymic stromal lymphoprotein
SEB	*S. aureus* enterotoxin type B
ROS	Reactive oxygen species
ER	Endoplasmic reticulum
LTA	Lipoteichoic acids
NET	Neutrophil extracellular trap
PKC	Protein kinase C
LPS	Lipopolysaccharide
ZO-1	Zona occludens-1
Hla	Alpha-hemolysin
pHNECs	primary human nasal epithelial cells
TSLP	Thymic stromal lymphopoietin
PCR	Polymerase Chain Reaction
RT-PCR	Real Time Polymerase Chain Reaction
ELISA	Enzyme Linked Immuno Sorbent Assay
EPSs	Extracellular polymeric substances
FESS	Functional endoscopic sinus surgery
ESS	Endoscopic sinus surgery
ECC	Estrogen containing contraception
AQP5	Aquaporin 5
PM	Particulate matter
URT	Upper respiratory tract
TRAP	Traffic-related air pollution
HPA	Hypothalamic–pituitary–adrenal
HRT	Hormone replacement therapy
SNMT	Sinonasal microbiota transplantation
EMT	Epithelial–mesenchymal transition
BCs	Basal cells
TJs	Intercellular junctions
DSG1/2	Desmoglein 1/2
GCS	Glucocorticosteroid
CFTR	Cystic fibrosis transmembrane conductance regulator
T2R	Type 2 taste receptor
NO	Nitric oxide
PCD	Primary ciliary dyskinesia
DNAH5	Dynein axonemal heavy chain 5
DNAI1	Dynein axonemal intermediate chain 1
AOAH	Acyloxyacyl hydrolase
(m)NGS	(metagenomic) Next-Generation Sequencing
NOS	Nitric oxide synthase
LAMA2	Laminin subunit alpha 2
LAMB1	Laminin subunit beta 1
MMPs	Matrix metalloproteinases
PAI-1	Plasminogen activator inhibitor-1
